# Evolutionary analysis and functional characterization of *BZR1* gene family in celery revealed their conserved roles in brassinosteroid signaling

**DOI:** 10.1186/s12864-022-08810-3

**Published:** 2022-08-08

**Authors:** Chunliu Zuo, Lan Zhang, Xinyue Yan, Xinyue Guo, Qing Zhang, Songyang Li, Yanling Li, Wen Xu, Xiaoming Song, Jinpeng Wang, Min Yuan

**Affiliations:** College of Life Sciences, North China University of Science and Technology, Tangshan, 063210 Hebei China

**Keywords:** Brassinosteroids, BZR1, Celery, Constitutive BR-responsive phenotypes, Whole-genome duplication

## Abstract

**Background:**

Brassinosteroids (BRs) are a group of essential steroid hormones involved in diverse developmental and physiological processes in plants. The Brassinazole-resistant 1 (BZR1) transcription factors are key components of BR signaling and integrate a wide range of internal and environmental signals to coordinate plant development, growth, and resistance to abiotic and biotic stresses. Although the *BZR1* family has been fully studied in *Arabidopsis*, celery *BZR1* family genes remain largely unknown.

**Results:**

Nine *BZR1* genes were identified in the celery genome, and categorized into four classes based on phylogenetic and gene structure analyses. All the BZR1 proteins shared a typical bHLH (basic helix-loop-helix) domain that is highly conserved across the whole family in Arabidopsis, grape, lettuce, ginseng, and three Apiaceae species. Both duplications and losses of the *BZR1* gene family were detected during the shaping of the celery genome. Whole-genome duplication (WGD) or segmental duplication contributed 55.56% of the *BZR1* genes expansion, and the γ as well as celery-ω polyploidization events made a considerable contribution to the production of the *BZR1* paralogs in celery. Four AgBZR1 members (AgBZR1.1, AgBZR1.3, AgBZR1.5, and AgBZR1.9), which were localized both in the nucleus and cytoplasm, exhibit transcription activation activity in yeast. AgBZR1.5 overexpression transgenic plants in Arabidopsis showed curled leaves with bent, long petioles and constitutive BR-responsive phenotypes. Furthermore, the *AgBZR1* genes possessed divergent expression patterns with some overlaps in roots, petioles, and leaves, suggesting an extensive involvement of *AgBZR1s* in the developmental processes in celery with both functional redundancy and divergence.

**Conclusions:**

Our results not only demonstrated that *AgBZR1* played a conserved role in BR signaling but also suggested that *AgBZR1* might be extensively involved in plant developmental processes in celery. The findings lay the foundation for further study on the molecular mechanism of the *AgBZR1s* in regulating the agronomic traits and environmental adaptation of celery, and provide insights for future BR-related genetic breeding of celery and other Apiaceae crops.

**Supplementary Information:**

The online version contains supplementary material available at 10.1186/s12864-022-08810-3.

## Introduction

Brassinosteroids (BRs) are a class of essential steroid hormones regulating diverse developmental and physiological processes, including cell elongation, photomorphogenesis, stomata patterning, male fertility, crop yield increase, and plant resistance to environmental factors [1–4]. BR-insensitive and BR-deficient mutants show severe growth defects, such as extreme dwarfism, delayed flowering, dark green leaves, reduced seed germination, and photomorphogenesis in darkness. The BR molecules are bound by a leucine-rich repeat receptor kinase named BRI1 (Brassinosteroid Insensitive1) [5, 6]. BRs binding enables heterodimerization, transphosphorylation and full activation of BRI1 with its co-receptor BAK1 (BRI1-Associated Receptor Kinase 1) to initiate downstream BR signaling [7–10]. Subsequently, a cascade of phosphorylation and dephosphorylation reactions are triggered to regulate the activities of downstream kinases and phosphatases. Eventually, this phosphorylation-mediated signal transduction activates two transcription factors, named BZR1 and BZR2/BES1 (*bri1*-EMS Suppressor 1), which bind to the BRRE (BR-response element) and/or E-box motifs of targets to regulate the expression of a variety of BR-responsive genes [11–14].

BZR1/2 are considered to be key components in BR signaling, which are phosphorylated by GSK3-like kinase BIN2 (Brassinosteroid-Insensitive 2) in the absence of BRs, and could not modulate the transcription of downstream target genes [15, 16]. BR signaling induces the dephosphorylation and activation of BZR1/2 by PP2A (Protein Phosphatase 2A) to modulate the transcription of downstream target genes [17, 18]. Through associating with different partners, BZR1/2 integrates a series of growth, and environmental signals to coordinate plant development, growth and resistance to abiotic and biotic stresses [19]. The *BZR1/2* family includes four additional homologs *BEH1* to *BEH4* (*BES1/BZR1* Homolog 1 to 4). A hextuple mutant (*bzr-h*) with disruption of the six *BZR1* family members displayed severe vegetative growth defects, which were very similar to the triple mutant of the BRs receptor BRI1 (*bri-t*) in Arabidopsis, indicating that the BZR1 family members are key transcription factors to govern BR-responsive gene expression [20]. Notably, two unusual β-Amylase-like proteins were found in Arabidopsis, which possess a C-terminal glucosyl-hydrolase domain in addition to the N-terminal well-defined BZR1-type domain. These two BZR1-BAM proteins (AtBAM7 and AtBAM8) bind to a G box cis-acting element and perform inverse functions to BZR1 on the expressions of target genes. The BZR1-BAM proteins may respond to sugars and mediate cross-talks between BR and metabolic signals [21].

*BZR1* orthologous genes play critical roles in plant growth and development by integrating BR and other signal pathways in many monocots and dicots species, indicating functional importance and conservation of the *BZR1* family among different species [22–25]. However, the genome-wide identification, evolutionary and functional analysis of the *BZR1* genes in celery (*Apium graveolens*) has yet to be performed, of which the whole-genome sequencing data has been released recently [26, 27]. Celery is a globally grown vegetable, and more than one part is edible, including crisp petiole, hypocotyl, leaf, and seed. Additionally, celery is also famous for its fragrance and medicinal value. It is rich in many pharmacologically active compounds, such as flavonoids, unsaturated fatty acids, volatile oils, and coumarins, which play essential roles in defending against pathogens. Exploring the *BZR1* family in the genome-wide might lay a foundation for further study on BR signaling, and provide insights to improve the agronomic traits in the breeding of celery and other Apiaceae crops.

## Results

### Identification and chromosomal location of *BZR1* genes

**Fig. 1:**
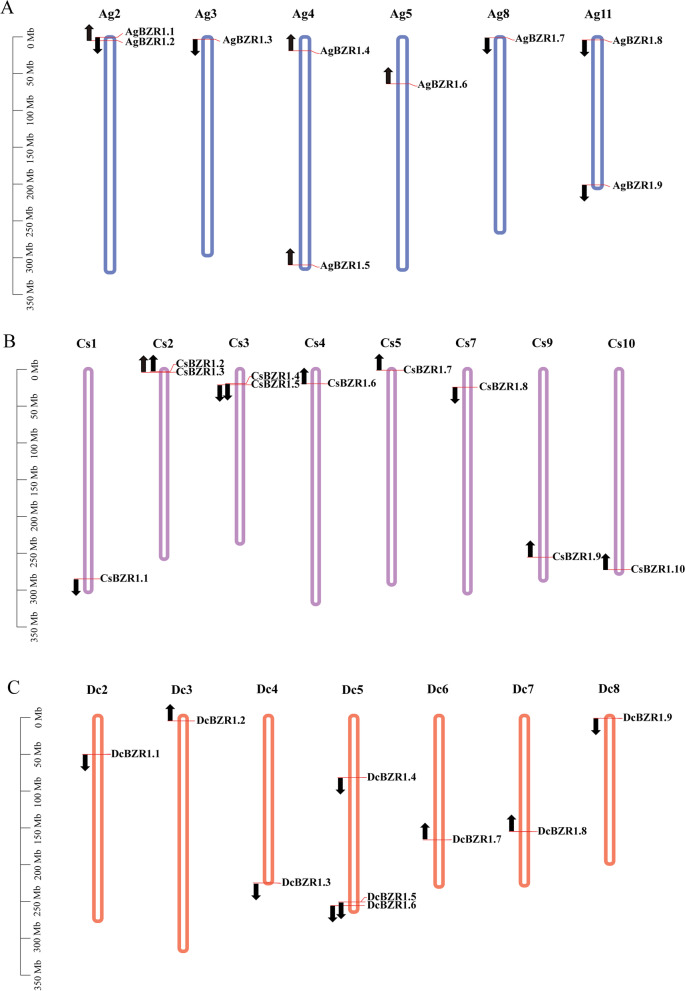
Chromosomal distribution of the *BZR1* genes in three Apiaceae species. Chromosomal distribution of the *BZR1* genes in celery **A**, coriander **B**, and carrot **C**. Red lines and arrows indicate the position and the direction of the *AgBZR1* genes, respectively. Celery (Ag), Coriander (Cs), Carrot (Dc)

Using AtBZR1 protein sequences as BLASTP queries, we identified 9, 10, and 9 *BZR1* genes in the genomes of celery, coriander, and carrot three Apiaceae species, respectively. Due to ginseng and lettuce showing the closest relationship with Apiaceae, and grape preserving the genome structure of dicots’ ancestor, those three genomes have also been screened for the *BZR1* gene family. A total of 20, 11, and 7 *BZR1* genes were identified in ginseng, lettuce, and grape, respectively. These *BZR1* genes were subsequently renamed according to their genomic locus (Fig. 1, Additional file 1: Table S1).

In celery, nine *AgBZR1* genes were unevenly located at six chromosomes, and most of them were located at the distal ends of chromosomes. Chromosomes 2 and 11 harbored two *AgBZR1* genes, while chromosomes 3, 4, 5, and 8 harbored one *AgBZR1* gene, respectively. Five *AgBZR1* genes were localized in a forward direction, while the other four members localized in a reverse direction.

### Phylogenetic and gene structure analysis of *BZR1* genes

**Fig. 2:**
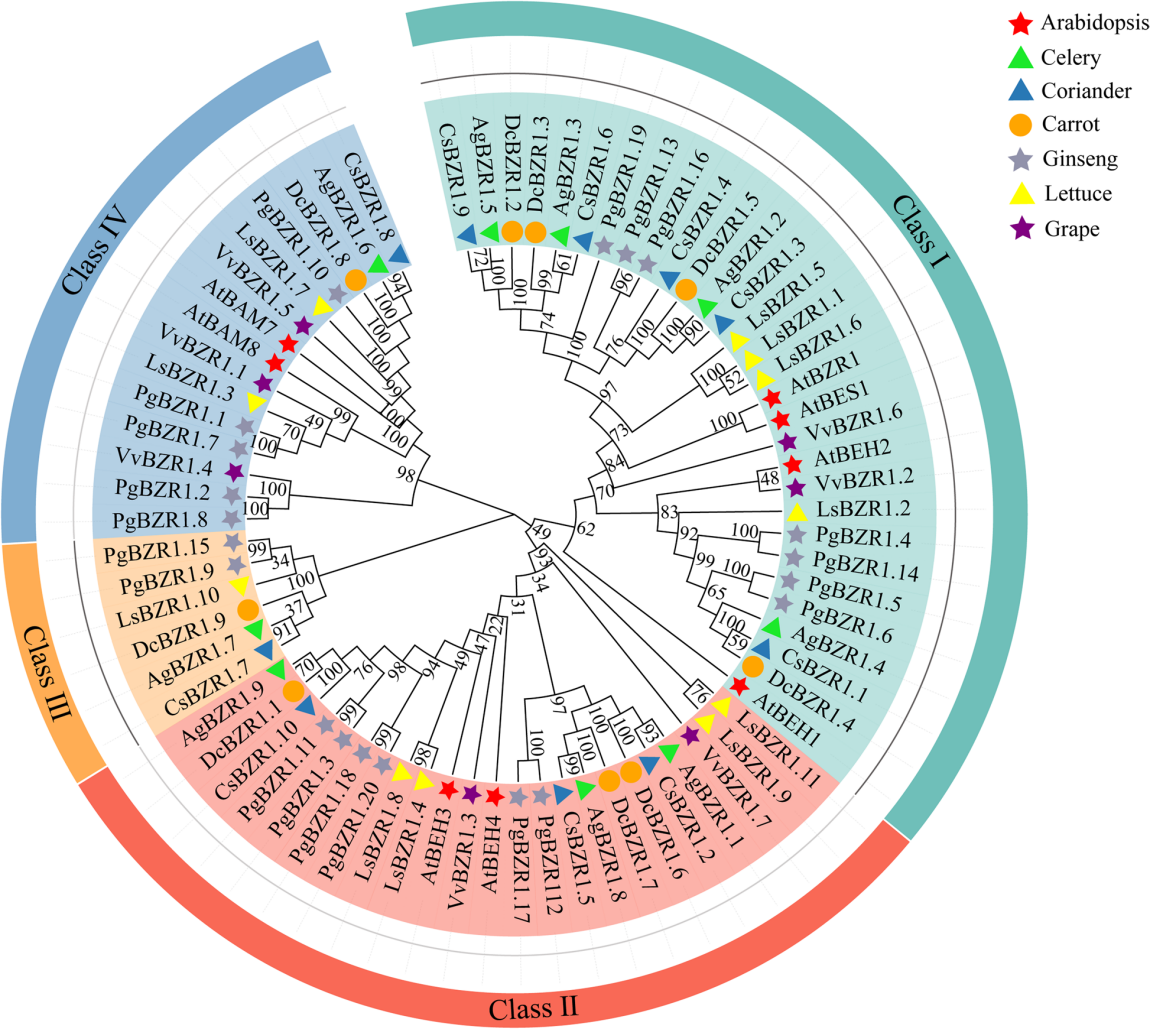
Phylogenetic analysis of the BZR1 proteins in the seven selected species. Classes I-IV were distinguished by distinct colors, and the bootstrap values were provided near nodes. Celery (Ag), Coriander (Cs), Carrot (Dc), Lettuce (Ls), Ginseng (Pg), Grape (Vv), and Arabidopsis (At)

**Fig. 3:**
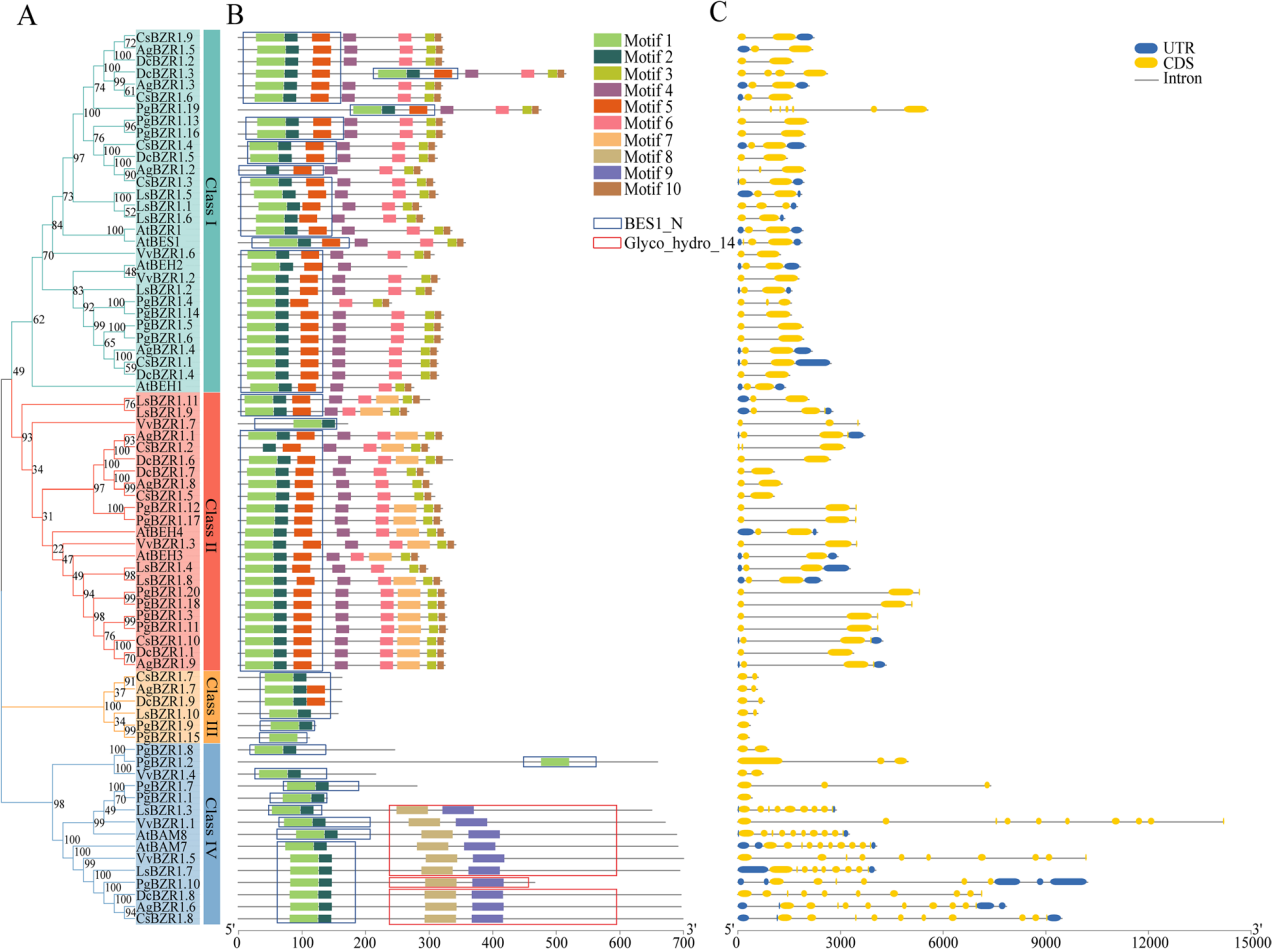
Conserved motifs and gene structure analyses of the *BZR1* gene family. **A** Phylogenetic tree of the BZR1 proteins from the seven selected plants. **B** Conserved motifs and domains of the BZR1 proteins. Different color boxes indicated different kinds of motifs. The positions of domains were framed by rectangular boxes. **C** Structures of the *BZR1* genes. The coding sequences (CDS) and untranslated regions (UTR) were displayed with yellow and blue boxes, respectively. The lines between boxes indicated introns

To explore the evolutionary history and phylogenetic relationship of the *BZR* gene family, we built a phylogenetic tree using the amino acid sequences of 74 BZR1s from the seven selected species, including celery, coriander, carrot, ginseng, lettuce, grape, and Arabidopsis (Fig. 2). Based on the phylogenetic analysis, all the BZR1s were categorized into four classes (I to IV) according to the classification and topology in Arabidopsis. Class I contained the largest number of BZR1s (30), followed by Class II (23) and Class IV (15). Class III (6) contained the fewest BZR1s (6). The key BR signaling transcriptional factors AtBZR1 and AtBES1 were clustered in Class I, with four homologs AgBZR1.2, AgBZR1.3, AgBZR1.4, and AgBZR1.5 in celery. The predominance of BZR1s in Classes I and II may indicate that regulating BR signaling is the primary function of this gene family. All the BZR1s shared a conserved bHLH (basic helix-loop-helix) BZR1-N domain, which functions as a typical DNA-binding domain. The BZR1s in Class IV concurrently possessing both the BZR1-N and glucosyl-hydrolase (Glyco_hydro_14, also named BAM-like) domains indicated that they may experience extra functions or function-combinations during evolution. Several ginseng BZR1s lack the BAM-like domain may be due to the ginseng genome data being on the scaffold level. Moreover, each of the three Apiaceae species contributed at least one BZR1 to these four groups, implying that the *BZR* gene family harbors a conservative evolutionary trend in Apiaceae species (Fig. 3).

### Conserved motifs analysis of BZR1s

To further characterize BZR1 proteins, conserved motifs were examined, and ten motifs were defined. The BZR1s in Class II harbored the most eight motifs, while those in Class III had the fewest two or three motifs. The motifs 1, 2, and 5 corresponded to the conserved BZR1-N domain, and a nuclear localization sequence (NLS) was localized in the motif 1. The motif 5, a serine-rich motif, was considered the phosphorylation region of GSK3 kinases. Among the nine AgBZR1s, AgBZR1.2, and AgBZR1.6 lack the motifs 1 and 5, respectively. Notably, some motifs were found to be group-specific. The motifs 4, 6, and 10 were specific for the BZR1s in Classes I and II. The partial motif 4 was the 14–3-3 binding motif, and the binding by 14–3-3 proteins retained the BZR1 proteins in the cytoplasm. The motif 6 was a PEST (proline-, glutamic-acid-, serine-, and threonine-rich) domain that putatively involved in protein degradation and protein phosphatase 2A dephosphorylation. The partial motif 10 corresponded to the EAR motif, which could mediate transcriptional regulation in the BZR1 and several other plant transcription factors. The motifs 8 and 9 were specific for the class IV and corresponded to BAM-like domain, which is characteristic of proteins associated with starch breakdown. Consequently, the BZR1s in this group possessed both the BZR1-N and BAM-like domains and had distinct functional annotations from the other three groups, which implying they may perform extra functions (Fig. 3A-B, Additional file 1: Table S2).

### Gene duplication and loss analysis of *BZR1* genes

**Fig. 4:**
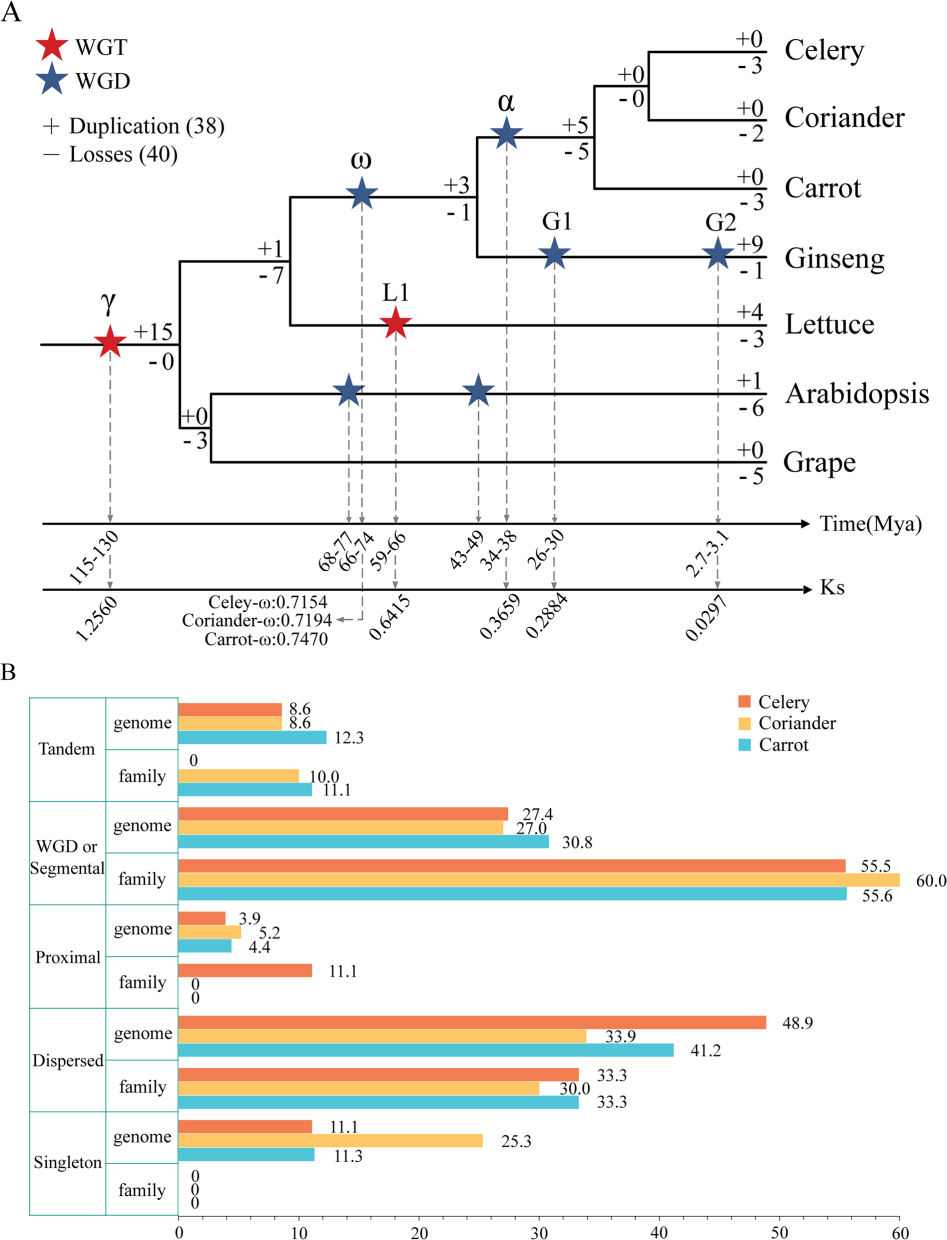
Duplication and loss analyses of the *BZR1* genes in three Apiaceae species. **A** Duplication and loss of the *BZR1* genes. The “ + ” and “–” symbols with values indicated gene duplication and loss, respectively. The lines below showed the time and Ks values corresponding to each polyploidization event. **B** Contribution of different duplication types to the *BZR1* duplicated gene pairs was assessed by calculating the percentage at the family and genomic scales, respectively

To investigate the evolutionary process of the *BZR1* gene family, we performed a comparative analysis among the seven selected genomes. The duplication and loss of the *BZR1* genes were examined in the evolutionary process. Interestingly, five *BZR1* genes were duplicated while five genes were lost (5 vs. 5) in the common ancestor of celery, coriander, and carrot, and even no duplications and losses were detected in the common ancestor of celery and coriander (0 vs. 0), which indicating high evolutionary conservation of the *BZR1* genes in celery and coriander. Remarkably, 3, 2, and 3 genes were lost in celery, coriander, and carrot, respectively, and no genes were gained in the three Apiaceae species, indicating that in addition to polyploidy events, the evolution of Apiaceae *BZR1* genes was also affected by other factors after the α WGD event. By contrast, more gene duplications were detected in ginseng due to two WGD events within species after divergence with Apiaceae species (Fig. 4A).

Subsequently, five gene duplication types, including WGD or segmental duplication, dispersed, tandem, singleton, and proximal, were examined to further analyze the expansion of the *BZR1* gene family. 55.56%, 60.00%, and 55.56% of the *BZR* genes expansion were contributed by WGD or segmental duplication, and consistently formed collinear blocks within each genome of celery, coriander, and carrot, respectively, implying the critical role of WGD or segmental duplication on the expansion of *BZR1* gene family in Apiaceae species (Fig. 4B, Additional file 1: Tables S3,S4, and S5).

### Collinearity analysis of *BZR1* genes

**Fig. 5:**
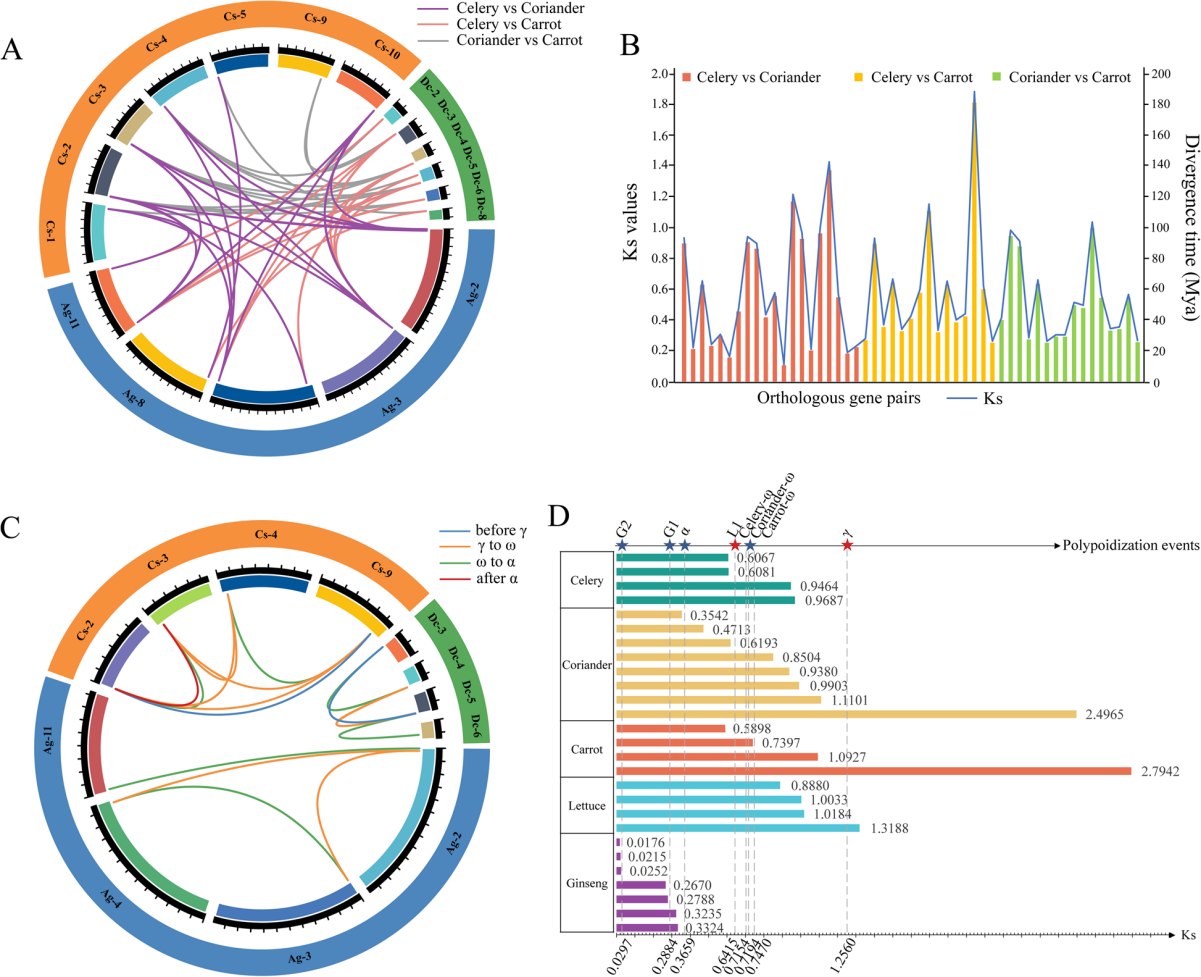
Analysis of the *BZR1* orthologous and paralogous pairs. **A** Circos plot of the *BZR1* orthologs in three Apiaceae species. **B** The Ks values and divergence times of the *BZR1* orthologous pairs between any two of the selected Apiaceae species. **C** Circos plot of the *BZR1* paralogs within each selected Apiaceae species. Different colored lines represented the *BZR1* paralogous pairs which were gained at different times according to the Ks values. **D** The appearance time of the *BZR* paralogous pairs was estimated by comparing the Ks values of collinear *BZR1* paralogous pairs with that corresponding to the polyploidization events. Columns in the bar chart indicated the Ks values of the *BZR1* paralogous pairs in the seven selected species, and the gray dotted lines indicated the Ks of the polyploidization events

Orthologous and paralogous gene pairs were explored to analyze the relationship of the *BZR1* genes between celery and other species. 20, 15, 12, 15, 6, and 7 *BZR1* orthologous pairs were detected between celery and coriander, carrot, ginseng, lettuce, grape, as well as Arabidopsis. The most *BZR1* orthologous pairs between celery and coriander proved the most recent evolutionary relationship between celery and coriander (Fig. 5A, Additional file 1: Table S6). The Ks (synonymous) values of collinear *BZR1* orthologous pairs were calculated, and the divergence time was estimated according to the Ks values of the *BZR1* orthologs between celery and other species. The celery *BZR1* orthologs diverged from that of Arabidopsis, coriander, and carrot approximately 44.69 to 88.36 Mya (Million years ago), 10.61 to 137.32 Mya, and 25.36 to 181.26 Mya, respectively (Fig. 5B, Additional file 1: Tables S7-S8). The Apiaceae *BZR1* orthologs diverged from that of Arabidopsis before and after the α WGD event, whereas the divergence within Apiaceae species occurred over a long-time span including the γ WGT event and two WGD events (ω/α), suggesting that some quite ancient *BZR1* orthologs were retained in three Apiaceae species due to conserved function.

Furthermore, the *BZR1* paralogous pairs were explored to analyze the relationship of the *BZR1* genes within species. 4, 8, and 4 paralogous pairs were detected within celery, coriander, and carrot, respectively. The most *BZR1* paralogous pairs (8) were found in coriander, which is consistent with fewer gene losses in coriander (Fig. 5C, Additional file 1: Table S9). To evaluate the impact of polyploidization on the *BZR1* paralogous pairs, the appearance time of the *BZR1* paralogous pairs was estimated by comparing the Ks values of the *BZR1* paralogous pairs with that corresponding to the polyploidization events (Additional file 1: Tables S10-S11). The Ks values of the γ (1.2560) and α (0.3659) polyploidization events were consistent in Apiaceae species, and were simply referred to as the γ and α events. While the Ks values of the ω event varied in celery (0.7154), coriander (0.7194), and carrot (0.7470), and thus referred to as the celery-ω, coriander-ω and carrot-ω event, respectively. Additionally, three specific polyploidization events were detected in lettuce and ginseng, and accordingly were named L1 (0.6415), G1 (0.2884), and G2 (0.0297) in this study. Moreover, the Ks of two celery *BZR* paralogous pairs (*AgBZR1.1*-*AgBZR1.8* and *AgBZR1.3*-*AgBZR1.5*, shown in green lines in Fig. 5C) were 0.6081 and 0.6067, and distributed in the 0.3659 < Ks < 0.7154 section corresponding to the α and celery-ω events. These results indicated that the two paralogous pairs might be produced accompanied by the celery-ω event in celery. Similarly, the Ks of another two celery *BZR* paralogous pairs (*AgBZR1.2*-*AgBZR1.3* and *AgBZR1.2*-*AgBZR1.5*, shown in yellow lines in Fig. 5C) were 0.9464 and 0.9687, and distributed in the 0.7154 < Ks < 1.2560 section corresponding to the celery-ω and γ events. The two paralogous pairs may be produced accompanied by the γ event. In conclusion, the γ and celery-ω polyploidization events played a leading role in the collinear *BZR1* paralogous pairs production in celery. In contrast, one collinear gene pair was retained in coriander after the α event, whereas all the collinear *BZR1* orthologous gene pairs in ginseng were gained after the ω event in Apiaceae, and 75% of the collinear *BZR1* orthologous pairs in lettuce was contributed by the γ event (Fig. 5D, Additional file 1: Tables S12-S13).

### Subcellular localization and transactivation activity analysis of AgBZR1 proteins

**Fig. 6:**
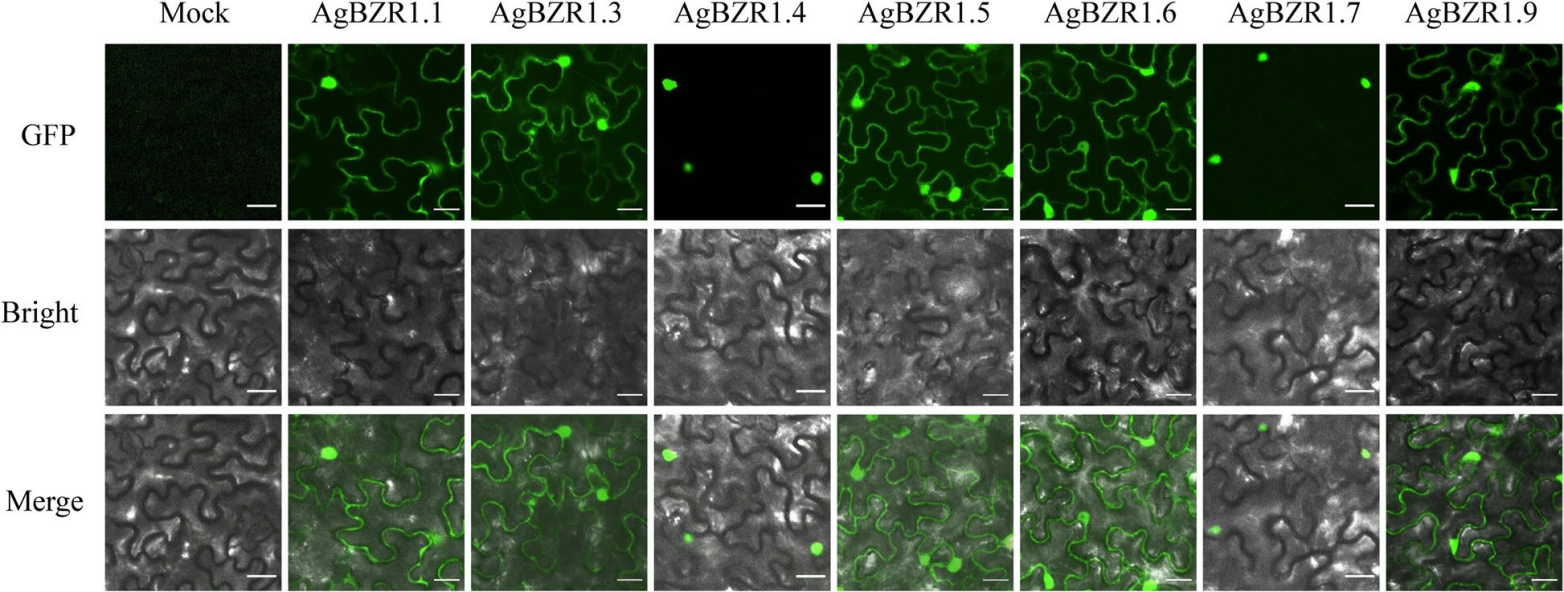
Subcellular localizations of the AgBZR1 proteins. Yellow fluorescence (YFP) signals were observed under the laser scanning confocal microscope to indicate the subcellular localizations of AgBZR1-YFP fusion proteins. Scale bars above represent 50 μm

**Fig. 7:**
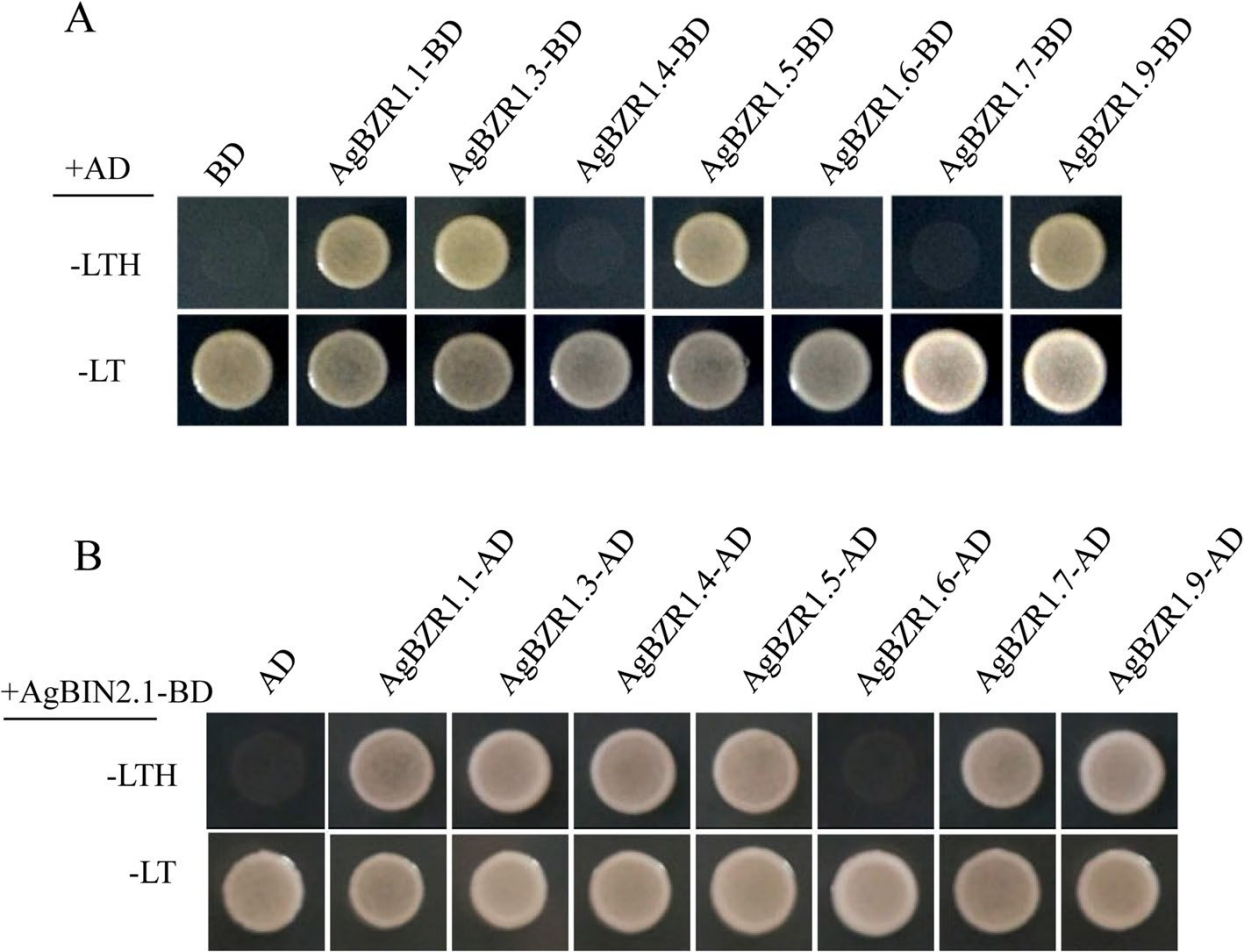
Transactivation activity and interaction analyses of the AgBZR1 proteins. **A** Transactivation activity analysis of the AgBZR1 proteins in yeast. The pGBKT7-*AgBZR1* and pGADT7 vectors were co-transformed into AH109 yeast cells. **B** AgBZR1 interacted with AgBIN2.1 in the yeast-two-hybrid assay. The pGADT7-*AgBZR1* and pGBKT7-*AgBIN2.1* constructs were co-transformed into AH109 yeast cells. The transformants were cultivated on the SD/-Leu-Trp (-LT) medium and screened on the SD/-Leu-Trp-His (-LTH) medium

Subcellular localization is crucial for gene functional research as it could indicate the working position of a protein at the cellular level. The protein sequence lengths of the AgBZR1s were predicted to vary from 162 aa (AgBZR1.1) to 696 aa (AgBZR1.6), and all the AgBZR1 proteins were predicted to be localized in nucleus (Additional file 1: Table S1). To verify the prediction of subcellular localization, YFP-fused AgBZR1 proteins were transiently expressed in *N. benthamiana* tobacco leaves. Except for *AgBZR1.2* and *AgBZR1.8* failure in cloning, five AgBZR1 proteins (AgBZR1.1, AgBZR1.3, AgBZR1.5, AgBZR1.6, and AgBZR1.9) were localized both in nucleus and cytoplasm, while two other AgBZR1 proteins (AgBZR1.4 and AgBZR1.7) were localized in nucleus alone (Fig. 6). As most of AgBZR1s were annotated as transcription factors, yeast-two-hybrid assays were conducted to detect the transactivation activity of AgBZR1 proteins. Yeast transformants harboring pGBKT7-AgBZR1.1, pGBKT7-AgBZR1.3, pGBKT7-AgBZR1.5, or pGBKT7-AgBZR1.9 grew well on the SD/ − Leu-Trp-His medium, indicating that the four AgBZR1 proteins possessing the nucleus and membrane dual localizations had transactivation activity. Conversely, yeast transformants harboring pGBKT7-AgBZR1.4 or pGBKT7-AgBZR1.7 didn’t grow on the SD/ − Leu-Trp-His medium, indicating that the two AgBZR1s with the single nucleus localization (AgBZR1.4 and AgBZR1.7) had no transactivation activity or had transcriptional repression activity, and both nuclear and cytosolic localization are important and necessary for AgBZRs performing transcriptional activation activity (Fig. 7A). Surprisingly, dually localized AgBZR1.6 had no transactivation activity, indicating that it may not perform transactivation function as a BAM-like protein.

BZR1 interacts with and is phosphorylated by the GSK3-like kinase BIN2 in BR signaling in Arabidopsis*.* The BIN2 orthologs were identified using AtBIN2 protein sequences as BLASTP queries (data not shown), and AgBIN2.1 with the highest protein identity was selected to test its interaction with AgBZR1s in the yeast-two-hybrid (Y2H) assay. Except for the BAM-like protein AgBZR1.6, all the other AgBZR1s interacted with AgBIN2.1 in yeast, implying that AgBZR1 also could be phosphorylated in celery, and AgBZR1.6 may not be functional as a transcriptional factor (Fig. 7B).

### Functional analysis of AgBZR1 in Arabidopsis

**Fig. 8:**
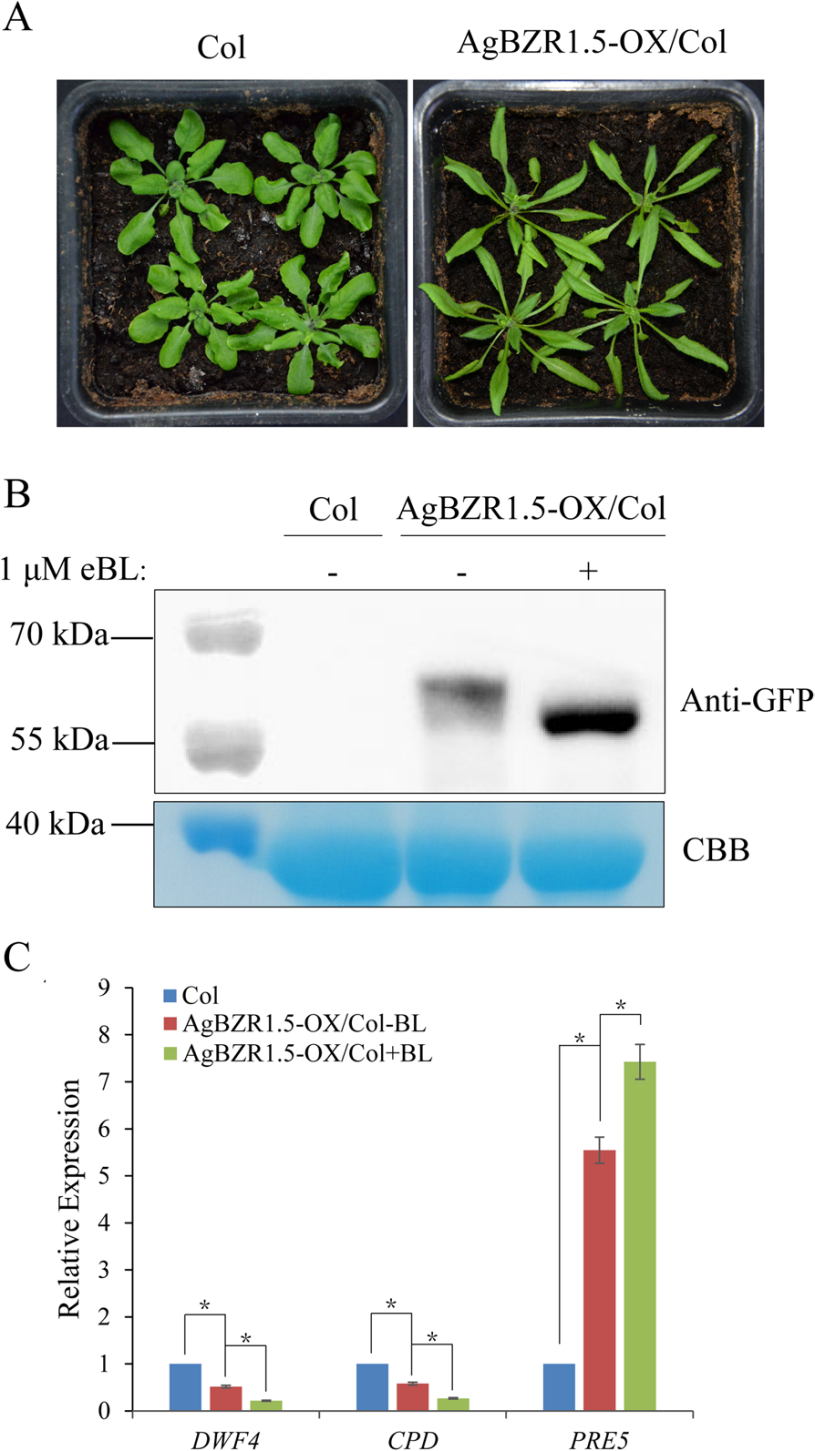
Plants overexpressing AgBZR1.5 exhibited BR-responsive phenotypes. **A** Phenotypes of light-grown 3-week-old Arabidopsis Col-0 overexpressing AgBZR1.5 with a YFP tag (AgBZR1.5-OX/Col). **B** The differential accumulation pattern of AgBZR1.5 protein was detected using an anti-GFP antibody. The upper and lower bands indicated the phosphorylated and unphosphorylated forms of AgBZR1.5 proteins, respectively. CBB (Coomassie brilliant blue G-250) staining was used to assess protein loading. Full-length blots/gels were presented in supplementary Fig. S1. **C** The expression analysis of BR-repressed genes (*DWF4* and *CPD*) and the BR-induced gene (*PRE5*) in the leaves of 3-week-old AgBZR1.5-OX/Col transgenic Arabidopsis plants under 1 μM eBL immersion for 1 h. Asterisk (*) denotes *P* < 0.05, as determined by a Student’s t-test

The AtBZR1/BES1 are critical transcriptional factors in BR signaling in Arabidopsis. AgBZR1.5 was clustered in the same group and shared the highest protein identity with AtBZR1/BES1. To verify whether AgBZR1s regulate BR responses, a YFP (yellow fluorescent protein) fusion to AgBZR1.5 encoding sequence under the control of CaMV 35S promoter was introduced into *Arabidopsis Columbia*-0 (Col-0). AgBZR1.5 with the fusion of YFP was successfully overexpressed in Col-0, and the overexpression transgenic plants (AgBZR1.5-OX/Col) exhibited quite a similar phenotype to the gain-of-function mutant *bes1-D* in *A. thaliana*, curled leaves with bent and long petioles (Fig. 8A). BRs induce the dephosphorylation of BZR1, which is considered a hallmark of active BR signaling pathway. Therefore, we detected whether AgBZR1.5 would respond to BR treatment in different lines of the transgenic plants. The results showed that AgBZR1.5 protein was predominantly phosphorylated (upper band) before BR treatment, and obviously dephosphorylated (lower band) upon BR treatment (Fig. 8B). Furthermore, the relative expression levels of the BR-induced gene (*PRE5*) and BR-repressed genes (*DWF4* and *CPD*) were evaluated using qRT-PCR assays to verify that AgBZR1.5 can regulate the expressions of target genes in BR signaling. Overexpression of AgBZR1.5 significantly increased the expression of *PRE5* and decreased the expressions of *DWF4* and *CPD*, and the change was significantly enhanced by eBL (active BRs) treatment, indicating that AgBZR1.5 causes a constitutive BR response phenotype (Fig. 8C). Overall, the results implied that AgBZR1 played important roles in BR signaling in celery.

### Function prediction through tissue-specific expression profiles of *AgBZR1* genes

**Fig. 9:**
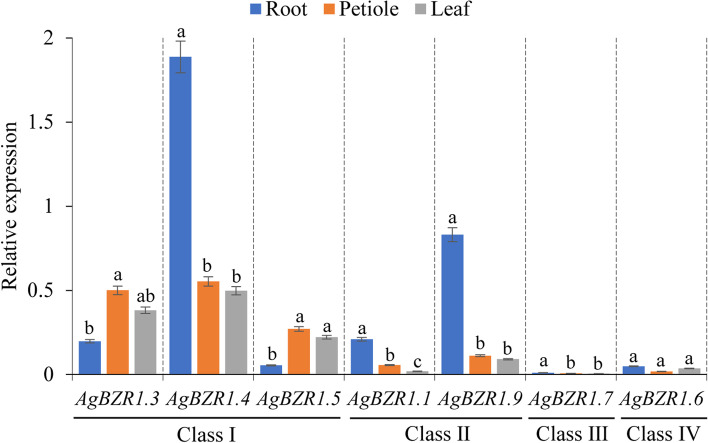
Tissue-specific expression patterns of the *AgBZR1* genes (root, petiole, and leaf) were determined by qRT-PCR. Small letters among different tissues for each gene indicate significant differences in the relative expression following Duncan’s test (*P* < 0.05), while the same letters represent no significant difference

The tissue-specific expression patterns usually implied the potential function of genes. Since the *BZR1* gene family integrates a wide range of hormonal, growth, and external signals to coordinate plant development and resistance to abiotic and biotic stresses in Arabidopsis, the functions of the *BZR1* orthologs in celery were predicted through tissue-specific expression profiling. The mRNA levels of *AgBZR1s* in different celery tissues were examined to explore tissue specificity. In general, compared to exceptionally low expression of the *AgBZR1s* from Classes III and IV (*AgBZR1.6* and *AgBZR1.7*), the *AgBZR1s* from Classes I and II were expressed ubiquitously in all the detected tissues, indicating an essential function of the *AgBZR1s* from Classes I and II and the functional divergence of *AgBZR1s* among different classes. Additionally, the expression patterns of the *AgBZR1s* in different classes differed with some overlap. The *AgBZR1* genes in Class II (*AgBZR1.1* and *AgBZR1.9*) were the most strongly expressed in roots and weakly expressed in petioles and leaves, implying a higher requirement for them in roots. The expression patterns of the *AgBZR1* genes in Class I showed a certain preference in different tissues. *AgBZR1.3* and *AgBZR1.5* exhibit similar expression patterns, with significantly lower expression in roots than that in petioles and leaves, implying potential function redundancy. In contrast, *AgBZR1.4* was expressed preferentially in roots. (Fig. 9). The results suggested that *AgBZR1s* were extensively involved in the developmental processes in celery with both function redundancy and divergence among different members.

## Discussion

Studies of several important crops and vegetables indicate that BRs play important roles in regulating plant growth, plant resistance, and productivity or fruit quality-related traits. However, BR signaling in celery remains uncharacterized. Identifying key components of BR signaling, understanding their evolution, and investigating their functions are considered to be critical steps for exploring BR signaling networks and initial stages for future BR-related genetic breeding in more crops and vegetables. To date, there has been no comprehensive study of the *BZR1* gene family in celery, which is a widespread and important vegetable plant. In the current study, we conducted a genome-wide analysis of the *AgBZR1* gene family, including gene identification, chromosomal location, phylogenetic relationship, gene structure, collinearity, as well as gene duplication and loss analyses. We further conducted a functional examination of this gene family, including subcellular localization, transcriptional activation activity, tissue-specific expression profiling, and responsiveness to BR treatment of AgBZR1 in Arabidopsis.

### Polyploidization events made a considerable contribution to the *BZR1* paralogous pairs in celery

Polyploidization, such as whole-genome duplications (WGD) or whole-genome triplications (WGT), is thought to be a process of large-scale genomic rearrangements and is often followed by genome modifications such as chromosome rearrangement, gene conversion, gene loss, and gene divergence of expression. Gene duplications often result from polyploidization, which supplies raw genetic materials and enables paralogous genes to undergo conservation, specialization, neo-functionalization, or sub-functionalization [28, 29].

Song, et al. reported that the celery genome had undergone the γ WGT in the eudicot ancestor and two sequential WGDs in Apiaceae, which resulted in the emergence of the celery genome structure. Subsequently, the majority of duplicated genes are lost [26, 30]. We then examined the impact of those duplication events on the *BZR1* genes in celery by using grape, Arabidopsis, lettuce, ginseng, and two other Apiaceae species (carrot and coriander) as references. Since the celery genome has undergone the ω and α two rounds of WGDs, whereas grape preserved the genome structure of dicots’ ancestor, one grape gene would correspond to four celery orthologs if there were no gene loss and translocation. There were supposed to be 28 *BZR1* genes based on 7 *BZR1* genes in grape. In fact, only 9 *BZR1* genes were detected in celery, which was consistent with that both duplications and losses of the *BZR1* gene family were detected in Fig. 4A. Collinearity analysis revealed that 55.56% of the *AgBZR1* genes were detected in collinear blocks, which was quite higher than the average level of the whole celery genome (27.43%) (Additional file 1: Table S5), suggesting that WGD made a bigger impact on the expansion of *AgBZR1* or higher retention of *AgBZR1* duplicated genes occurred after WGD. The findings were consistent with the report that the retention of duplicated genes is usually biased, and the genes with critical functions such as kinases/phosphatase, transcription factors, transporters, as well as the genes in transcriptional regulation networks tend to be preferentially retained after WGDs [31–35]. The tissue-specific expression analysis in this study revealed that the *AgBZR1* genes possessed divergent expression patterns, which may be greatly helpful for gene retention after WGDs (Fig. 9). However, small-scale gene duplications could occur and result in gene dosage imbalanced. Certain evolutionary strategies might be employed by different gene families to retain the duplicated genes and keep the gene dosage balanced. For example, the highly specific interaction and divergent gene expression pattern jointly maintained the balanced dosage for the CBL (Calcineurin B-like protein) and CIPK (CBL-interacting protein kinase) interacting partners [36]. We found that the expansion of the *AgBZR1* gene family also was contributed by dispersed and proximal duplications. The *BZR1* gene family, as key transcription factors in BR signaling, forms a regulation network together with thousands of BR-responsive genes to mediate plant growth, development, and stress response. Whether certain evolutionary strategies exist between BZR1 and the target genes in the transcriptional network to maintain gene dosage balanced requires more detailed study in the future.

### AgBZR1 proteins performed important functions in celery

In *Arabidopsis thaliana*, all the eight BZR1/BES1 family members have an N-terminal bHLH DNA binding motif that is potentially responsible for DNA binding as transcriptional factors, whereas the functions of those BZR1 homologs have diverged. BZR1 represses the expression of BR-biosynthetic genes through binding to BR-response elements (BRRE, CGTGC/TG), while BES1 is demonstrated to activate gene expression via binding to the E box motif (CANNTG) [11, 37]. The AtBAM7 and AtBAM8 bind to similar cis-elements of target genes but perform opposite functions in comparison to BZR1 on gene expression [21]. In this study, nine *BZR1* genes were identified in celery and divided into four classes based on a phylogenetic tree with the distinct evolutionary relationships. Gene structure analysis revealed that all the AgBZR1 proteins possessed a conserved BZR1-N domain and an NLS sequence across the whole family. Subsequently, subcellular localization and transcriptional activity analyses showed that all the AgBZR1s exhibited nuclear localization, and five members (AgBZR1.1, AgBZR1.3, AgBZR1.5, AgBZR1.6, and AgBZR1.9) exhibited nuclear and cytosolic dual localizations. Furthermore, the four members (AgBZR1.1, AgBZR1.3, AgBZR1.5, and AgBZR1.9) with nuclear and cytosolic dual localizations showed the transcriptional activation activity, implying that both nuclear and cytosolic localizations are important and necessary for AgBZR1s performing transcriptional activation activity. Particularly, AgBZR1.6, the closest homolog of two Arabidopsis β-amylase-like proteins (AtBAM7 and AtBAM8), didn’t show the transcriptional activation activity in yeast, although it possessed nuclear and cytosolic dual localizations. BAM7 and BAM8 are demonstrated to perform inverse transcription effects compared to BZR1 in Arabidopsis*.* We speculate that AgBZR1.6 may also undergo function differentiation and perform transcriptional repression activity instead of transcriptional activation activity. Additionally, thousands of nonfunctional duplicates existing in the plant genome, are referred to as pseudogenes without expression, and only a small subset of pseudogenes may still be functional as truncated proteins. Since *AgBZR1.2* and *AgBZR1.8* failed in cloning in this study, a further investigation is required to confirm whether they are pseudogenes.

In Arabidopsis, BZR1 is phosphorylated by BIN2 in the absence of BR, and the phosphorylated BZR1 will be dephosphorylated upon BR signaling. Motif analysis revealed that the motif 5 correspond to the phosphorylation sites of GSK3 kinases, and only AgBZR1.6 distributed in Class IV lacked this motif. Consistently, except for AgBZR1.6, all the other AgBZR1 proteins were demonstrated to interact with AgBIN2.1 in yeast, which implied the conserved functions of AgBZR1 proteins in BR signaling and AgBZR1 proteins could also be regulated by the phosphorylation status in celery. Due to AgBZR1.5 possessed nuclear and cytosolic dual localizations and exhibited transcriptional activation activity, it was selected and transformed into *Arabidopsis Columbia*-0 (Col-0) under the control of CaMV 35S promoter (AgBZR1.5-OX/Col). Consistently, AgBZR1.5-OX/Col transgenic plants produced bent petioles and curled leaves similar to the gain-of-function mutant *bes1-D* in Arabidopsis. BZR1 and BES1 are demonstrated to be functional and stabilized in the dephosphorylated form [14, 18]. In the current study, eBL treatment induced AgBZR1.5 protein dephosphorylation, which implied the conserved functions of AgBZR1 and expanded our understanding of the BR response in celery.

BRs induce the inactivation of BIN2 and subsequent activation of the BZR1 transcriptional factors, which would be functional indispensably and redundantly in modulating the expression of downstream target genes in Arabidopsis. For example, BZR1 enhances plant freezing tolerance via regulating the expression of CBFs (C-repeat binding factor) [38]. BES1 binds to the promoter regions of CESAs (cellulose synthase genes) to regulate cellulose biosynthesis [39]. Meanwhile, the BZR1 transcriptional factors could also integrate a wide range of signal pathways through co-regulating target genes together with different partners in many monocots and dicots species. That may be the reason why BRs are extensively involved in multiple plant developmental and physiological processes, as well as plant resistance to environmental factors in Arabidopsis and many crop plants. For instance, BZR1/BES1 regulate hypocotyl elongation through integrating light and hormone signaling pathways together with the partner PIFs (Phytochrome-interacting Factors) in Arabidopsis [40]. GmBEHL1, a soybean *BZR1/BES1* homolog, integrates BR signaling with nodulation signaling to negatively regulate nodulation in soybean [41]. BR signaling coordinates sugar and hormonal signals through the BZR1 transcription factors to regulate bud outgrowth in tomato [42]. Based on the above findings, we speculate that the BZR1 transcription factors may also serve as integration nodes between BR and other signaling pathways to coordinate celery growth, development, and resistance to environmental factors. We then performed functional prediction through tissue-specific expression analysis of the *AgBZR1* genes in celery. The *AgBZR1* genes from different classes in the phylogenetic tree exhibit distinct expression patterns, suggesting the functional divergence of the *AgBZR1* genes among different classes. Meanwhile, similar expression patterns were detected among *AgBZR1.1*, *AgBZR1.4*, and *AgBZR1.9*, as well as between *AgBZR1.3* and *AgBZR1.5*, implying the potential functional redundancy. The molecular mechanism of the *AgBZR1s* in regulating celery growth, development, and environmental adaption requires to be further studied due to the limitation of celery transgenic technology at present.

## Conclusions

Overall, a comprehensive study of the *BZR1* gene family in celery, including both evolutionary and functional examinations, were conducted in this study. Nine *BZR1* genes were identified in the celery genome and categorized into four classes according to phylogenetic and gene structure analyses. The γ and celery-ω polyploidization events greatly contributed to the *BZR1* paralogous pairs. The results from subcellular localization, transactivation activity, and transgenic Arabidopsis indicated that AgBZR1 proteins performed important functions in BR signaling. Tissue-specific expression profiling revealed that the *AgBZR1* genes possessed divergent expression patterns with some overlaps, suggesting their extensive involvement in the developmental processes with both function redundancy and divergence in celery. Our findings lay the foundation for further study of *AgBZR1* function in celery.

## Materials and methods

### Identification of *BZR1* genes and phylogenetic analysis

To identify the *BZR1* gene family, the amino acid sequences of AtBZRs were used as bait sequences to query the corresponding genome sequences by BLASTP [43] with an e-value of 10^–5^. Furthermore, the Pfam (https://pfam.xfam.org/), SMART (http://smart.embl-heidelberg.de/), as well as CDD (https://www.ncbi.nlm.nih.gov/cdd/) databases were used to verify the conserved domains. The BZR1s amino acid sequences in the selected species were aligned by MUSCLE5.1. The phylogenetic tree was built using the maximum likelihood (ML) method with MEGA11 based on JTT model under 1,000 bootstrap replications.

### Chromosomal distribution

The chromosomal location of each *BZR1* gene, including the chromosome number, and starting and ending positions, were retrieved from the gff (general feature format) file. Furthermore, the chromosome mapping of the *BZR1* genes were built using Mapchart [44] software with default parameters.

### Gene structure and conserved motif analysis

GSDS was used to draw the structure of BZR1 proteins based on the position information of exons, introns, and UTRs from gff files. The online MEME tool (https://meme-suite.org/meme/) was employed to analyze the conserved motifs of BZR1 proteins with default parameters.

### Gene duplication and loss analysis and collinearity

The genomic data of the seven selected species were aligned by BLASTP with an e-value of 1e^−5^, then the alignment results were submitted to the MCScanX [45] software for the collinearity analysis. The collinear blocks of the corresponding species were identified, and then the collinear *BZR1* gene pairs were examined. Circos [46] was used to illustrate the relationships among the *BZR1* orthologs and paralogs. The gene duplication type was analyzed by duplicate_gene_classifier sub-program, including whole genome/segmental, tandem, proximal, dispersed, and singleton. Notung [47] was used to analyze the replication and loss of the *BZR* gene family during evolution by reconciling the species tree and the gene tree.

### Ks calculation and divergence time estimation

Coding sequences of the *BZR1* orthologous and paralogous pairs were aligned by MUSCLE [48], and the Ks values of the gene pairs were calculated by a python script we wrote based on the NG86 algorithm, respectively.

The divergence time (T) of the *BZR1* orthologous pairs was estimated by the equation T = Ks/2r. The “r” is the neutral substitution rate, which is 5.2 × 10^−9^ substitutions per site per year when calculating the divergence time within Apiaceae species, and is 1.5 × 10^–8^ substitutions per site per year (the substitution rate of dicotyledonous plants) when calculating the divergence time between celery and Arabidopsis [26].

The appearance time (AT) of the *BZR1* paralogous pairs was estimated by comparing the Ks values of the *BZR1* paralogous pairs to that of the polyploidization events within the seven selected species. The Ks of the polyploidization events within each species are shown in Table S12. The *BZR1* paralogous pairs were indicated with different lines based on different ATs. The blue, orange, green, and red lines represent the AT is before the γ event, before the ω event and after the γ event, after the ω event before the α event, as well as after the α event, respectively.

### Plant materials, growth conditions and BR treatment

The Arabidopsis *(Arabidopsis thaliana* Col-0), tobacco (*Nicotiana benthamiana* L.), and celery (*Apium graveolens*) plants were used in the AgBZR1s overexpression, subcellular localization, and tissue-specific expression profiling experiments, respectively. The plants were grown under 22 to 25 °C at a 16 h light:8 h dark cycle.

For BR treatment, eBL powder was dissolved in 80% ethanol to make the 1 mM stock solution. The leaves from the Arabidopsis plants at the 4-leaf stage were collected and soaked in ½MS medium containing 1 μM ebL for 1-h. eBL was instead by 80% ethanol in the control group. All samples were collected and ground in liquid nitrogen frozen for further immunoblotting analysis.

### Vector construction

Full-length cDNA of *AgBZR1s* were amplified by PCR and cloned into entry vectors (PBM27, Biomed), and then recombined with multiple gateway-compatible destination vectors pEarleygate104, PGADT7, and PGBKT7 to generate *p35S:AgBZR1s-YFP*, *GAL4AD-AgBZR1s*, and *GAL4BD-AgBZR1s* expression vectors. Oligo primers used are listed in the supplementary data (Additional file 1: Table S14).

### Subcellular localization of AgBZR1s proteins

The *p35S:AgBZR1s-YFP* expression vectors were transformed into *Agrobacterium tumefaciens* (GV3101), which harboring *p35S:AgBZR1s-YFP* the expression vectors were infiltrated into the leaf epidermal cells of *Nicotiana benthamiana*. After 36 h infiltration, the yellow fluorescence (YFP) signals were visualized under a laser scanning confocal microscope (Leica, Germany).

### Transactivation activity analysis

*AgBZR1s*-pGBKT7 and *AgBIN2.1*-pGADT7 constructs were co-transformed into AH109 yeast cells, which were cultivated on the SD/-Leu-Trp medium plates for transformants. Transactivation activity was determined by growing yeast transformants on the SD/-Leu-Trp-His medium plates.

### Immunoblotting

Arabidopsis leaves were ground to powder in liquid nitrogen. The total proteins were extracted in 2X SDS sample buffer, separated in the 8% SDS–PAGE gel, and blotted to the polyvinylidene fluoride (PVDF) membrane. Then the blots were probed with an anti-GFP primary antibody (Cat: G1002, LABLEAD, Beijing, China), followed by an HRP-conjugated secondary antibody (Cat: S0100, LABLEAD, Beijing, China). The membrane was stained with Coomassie brilliant blue G-250 (CBB) to assess protein loading.

### Quantitative real-time PCR (qRT-PCR)

Root, petiole, and leaf from 4-week-old celery plants were harvested, and total RNA of each tissue was extracted. Then the qRT-PCR assay was conducted to assess the expression patterns of *AgBZR1s* based on standard protocols. Three biological replicates were conducted for each *AgBZR1* in each tissue. The primers used in the current study are listed in Table S14, and the internal reference is the *AgTUB-B* gene of celery.

## Supplementary Information

**Additional file 1:** **Table S1.** Gene name and ID in the seven selected species. **Table S2.** The detailed information of the motifs in BZR proteins. **Table S3.** Types of duplications of BZR1 family genes in the seven selected species. **Table S4.** Types of duplications of BZR1 genes in celery, coriander and carrot. **Table S5.** Synteny analysis of BZR1 genes and all other genes in the genomes of the three Apiaceae species. **Table S6.** List of orthologous BZR1 gene pairs in celery, coriander and carrot. **Table S7.** Ka/Ks ratios and divergence time of orthologous BZR1 gene pairs in celery, coriander and carrot. **Table S8.** Ka/Ks ratios and divergence time of orthologous BZR1 gene pairs in celery and Arabidopsis. **Table S9.** List of paralogous BZR1 gene pairs in in celery, coriander and carrot. **Table S10.** Non-synonymous (Ka) and synonymous substitute rate (Ks) between syntenic gene pairs in the seven selected species. **Table S11.** Non-synonymous (Ka) and synonymous substitute rate (Ks) between syntenic gene pairs in celery, coriander and carrot. **Table S12.** Ks distribution related to duplication events within each genome. **Table S13.** The correlation between BZR1 paralog gene pairs retain and whole genome duplication events based on Ks values. **Table S14.** List of primers used in this study.

**Additional file 2:** **Fig. S1** Full-length blots/gels of Fig. 8B are presented.**A-E** indicated multiple exposure images. **F** indicated the CoomassieBrilliant Blue (CBB) staining result.

## Data Availability

The genome sequence information of the seven selected species were obtained from the following websites. Celery genome database (CGD): http://celerydb.bio2db.com/; Coriander genome database (CGDB): http://cgdb.bio2db.com/; Ginseng genome database: http://ginsengdb.snu.ac.kr/data.php; Genome sequences of carrot, lettuce, and grape from Phytozome database: https://phytozome-next.jgi.doe.gov/; Arabidopis TAIR database: https://www.arabidopsis.org/. The data sets supporting the conclusions of this study are included within the article and its additional files.
