# Supplementary material for: Evolutionary analysis and functional characterization of BZR1 gene family in celery revealed their conserved roles in brassinosteroid signaling
Source: BMC Genomics. 2022 Aug 8;23:568. doi: 10.1186/s12864-022-08810-3 (PMC9361572; doi:10.1186/s12864-022-08810-3)
Supplement: Supplementary file 2 — Additional file 2: Fig. S1 Full-length blots/gels of Fig. 8B are presented.A-E indicated multiple exposure images. F indicated the CoomassieBrilliant Blue (CBB) staining result. [file 12864_2022_8810_MOESM2_ESM.docx]

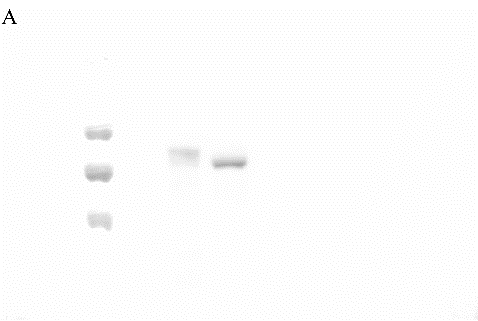

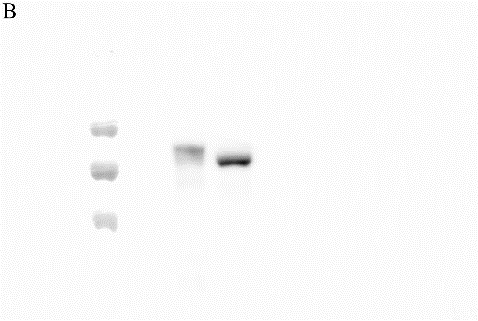


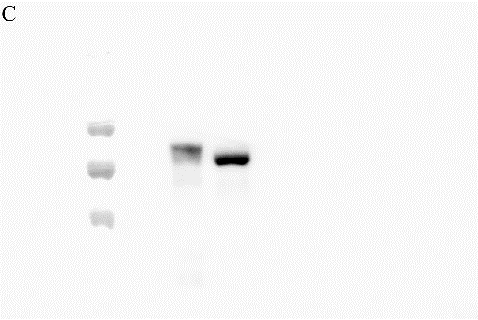

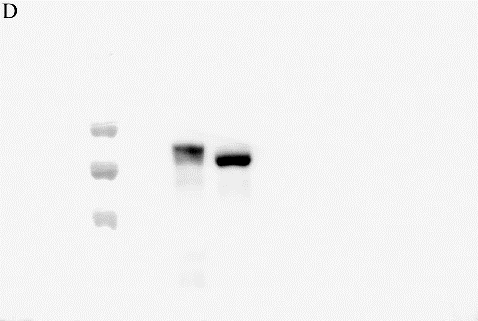


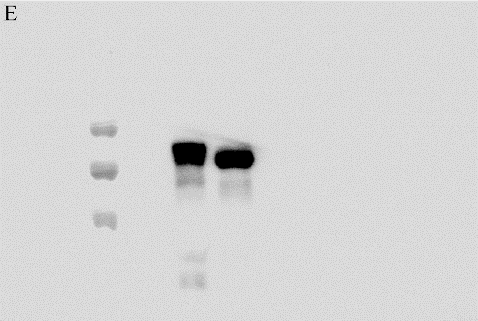

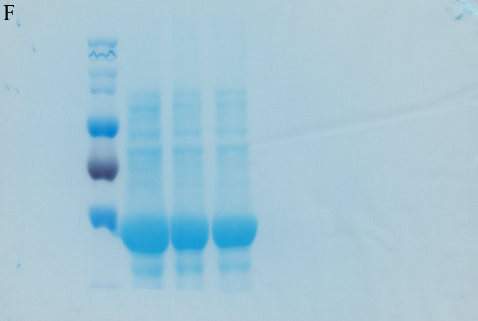


Fig. S1 Full-length blots/gels of Fig. 8B are presented. **A-E** indicated multiple exposure images. **F** indicated the Coomassie Brilliant Blue (CBB) staining result.
